# *DYNC2LI1* mutations broaden the clinical spectrum of dynein-2 defects

**DOI:** 10.1038/srep11649

**Published:** 2015-07-01

**Authors:** Kristin Kessler, Ina Wunderlich, Steffen Uebe, Nathalie S. Falk, Andreas Gießl, Johann Helmut Brandstätter, Bernt Popp, Patricia Klinger, Arif B. Ekici, Heinrich Sticht, Helmuth-Günther Dörr, André Reis, Ronald Roepman, Eva Seemanová, Christian T. Thiel

**Affiliations:** 1 Institute of Human Genetics, Friedrich-Alexander-Universität Erlangen-Nürnberg, Erlangen, Germany; 2 Animal Physiology, Friedrich-Alexander-Universität Erlangen-Nürnberg, Erlangen, Germany; 3 Department of Orthopaedic Rheumatology, Friedrich-Alexander-Universität Erlangen-Nürnberg, Erlangen, Germany; 4 Institute of Biochemistry, Friedrich-Alexander-Universität Erlangen-Nürnberg, Erlangen, Germany; 5 Department of Pediatrics and Adolescent Medicine, Friedrich-Alexander-Universität Erlangen-Nürnberg, Erlangen, Germany; 6 Department of Human Genetics, Radboud University Medical Center, Nijmegen, Netherlands; 7 Department of Clinical Genetics, Institute of Biology and Medical Genetics, 2nd Medical School, Charles University, Prague, Czech Republic

## Abstract

Skeletal ciliopathies are a heterogeneous group of autosomal recessive osteochondrodysplasias caused by defects in formation, maintenance and function of the primary cilium. Mutations in the underlying genes affect the molecular motors, intraflagellar transport complexes (IFT), or the basal body. The more severe phenotypes are caused by defects of genes of the dynein-2 complex, where mutations in *DYNC2H1*, *WDR34* and *WDR60* have been identified. In a patient with a Jeune-like phenotype we performed exome sequencing and identified compound heterozygous missense and nonsense mutations in *DYNC2LI1* segregating with the phenotype. *DYNC2LI1* is ubiquitously expressed and interacts with DYNC2H1 to form the dynein-2 complex important for retrograde IFT. Using *DYNC2LI1* siRNA knockdown in fibroblasts we identified a significantly reduced cilia length proposed to affect cilia function. In addition, depletion of *DYNC2LI1* induced altered cilia morphology with broadened ciliary tips and accumulation of IFT-B complex proteins in accordance with retrograde IFT defects. Our results expand the clinical spectrum of ciliopathies caused by defects of the dynein-2 complex.

The primary cilium is a nearly ubiquitous organelle of non-proliferative vertebrate cells. This organelle functions as an antenna to sense extracellular stimuli via various ciliary membrane receptors and transmits these signals into the cell to initiate intracellular transduction cascades of different signaling pathways^1^,^2^,^3^,^4^. These include the Hedgehog, Wnt, planar cell polarity, FGF, Notch, mTor, PDGF and the Hippo signaling pathways^5^,^6^,^7^,^8^,^9^,^10^,^11^,^12^,^13^.

Cilia play important roles in differentiation, migration, proliferation, determination of left-right asymmetry and are thus important for the embryonic and postnatal development and proper organ function in adulthood^1^,^14^,^15^. The non-motile primary cilium consists of two main components, the basal body complex on the cytoplasmic side of the cell membrane, and the ciliary axoneme, surrounded by a cytoplasmic membrane extending into the extracellular space. The axoneme is radially arranged by nine microtubule doublets growing out of the distal end of the basal body (9 × 2 + 0 structure). The basal body complex consists of a mother and daughter centriole embedded in the pericentriolar material (PCM)^3^,^4^,^14^,^16^,^17^. The junction between the basal body and the axoneme, the transition zone, is a specialized structure with a barrier function (“ciliary gate”), regulating the passage of ciliary proteins into or out of the primary cilium^18^,^19^.

During the cell cycle cells present a primary cilium in G0/G1 phase and before entering mitosis the cilium is disassembled. For assembly, maintenance and dismantling different proteins are required, brought to their destination via the intraflagellar transport (IFT). The IFT is a bidirectional transit system that transports cargos to the tip of the cilium (anterograde transport) and back to the base (retrograde transport)^17^,^20^,^21^. Kinesin-2 motors and subunits of the IFT-B complex are integral parts of the anterograde movement, whereas cytoplasmic dynein-2 and IFT-A components are responsible for retrograde transport^16^,^22^,^23^. Cell cycle regulatory signals, cytoplasmic vesicle transport systems and recruitment of all required IFT components at the right time and the right place are important for proper ciliogenesis.

Defects of genes encoding a variety of proteins involved in cilia formation, maintenance and function, such as IFT subcomponents and components of the ciliary axoneme, transition zone or basal body, lead to the broad phenotypic spectrum of ciliopathies^15^,^24^. Many of the associated phenotypes include brain malformations, polydactyly, kidney cysts, retinal degeneration, and skeletal abnormalities. Today, mutations in 14 different genes have been identified to be causative for skeletal ciliopathies. Because of the genetic heterogeneity of skeletal dysplasias, disrupted proteins affect either the dynein motor (DYNC2H1), or the core components of the IFT transport complexes (IFT43, IFT80, IFT122, IFT140, IFT172, WDR19, WDR34, WDR35, WDR60 and TTC21B) or the basal body region (NEK1, EVC, EVC2)^25^,^26^,^27^,^28^,^29^,^30^. The phenotypic spectrum of skeletal ciliopathies includes short ribs, narrow thorax, short stature, postaxial polydactyly and other skeletal abnormalities. These phenotypes are shared by the skeletal short-rib thoracic dysplasias (SRTD 1 - 12 [MIM 208500, 611263, 613091, 613819, 614376, 263520, 614091, 615503, 266920, 615630, 615633, 269860]), Ellis-van Crefeld syndrome (EVC [MIM 225500]), cranioectodermal dysplasias (CED1-4 [MIM 218330, 613610, 614099, 614378]) and Weyers acrofacial dysostosis (WAD [MIM 193530])^25^,^31^,^32^,^33^.

Here, we performed exome sequencing in a family with a Jeune-like intermediate phenotype (SRTD and EVC overlapping clinical findings) and identified compound heterozygous nonsense and missense mutations in the *DYNC2LI1* gene. DYNC2LI1 is known as a component of the dynein-2 complex important for retrograde IFT^34^,^35^. Knockdown experiments resulted in shorter cilia with abnormally bulged tips, similar to other ciliopathies with retrograde IFT defects. Mutations in the light intermediate chain of the dynein-2 complex are further expanding the clinical spectrum of skeletal ciliopathies.

## Results

### Clinical features of patients with *DYNC2LI1* mutations

The affected children were the offsprings of non-consanguineous parents (Supplementary Fig. 1a). The first child (patient 1) developed a respiratory distress syndrome and died at the age of 3 days. Three further pregnancies ended as missed abortions. The second child (patient 2) developed well, but showed dysmorphic features. During the following pregnancy fetal postaxial polydactyly was noticed and the pregnancy terminated at 19^th^ week of gestation (patient 3). The observed clinical features were classified between Ellis-van-Crefeld syndrome and Jeune syndrome. Although the affected children present with variable features they do share characteristics in common.

Patient 1 showed a polyhydramnion during pregnancy and was born at 38^th^ weeks of gestation with a weight of 3300 g (0.3 s.d.) and a body length of 50 cm (–0.17 s.d.). Clinical findings included agenesis of the epiglottis, postaxial hexadactyly of the both hands and the right foot, hypoplastic nails, atrioventricular septal defect and hepatosplenomegaly (Supplementary Fig. 1b-d). She presented with distinctive facial features characterized by medial cleft lip, low set ears, asymmetric tongue, hyperplastic gingivae, epicanthal fold and down-slanting palpebral fissures. A hypoplastic thorax led to a respiratory distress syndrome and the patient died two days after birth. Her karyotype was normal (46,XX).

Patient 2 also showed a polyhydramnion during pregnancy and was born at 40^th^ week of gestation with a weight of 4120 g (1.53 s.d.) and a body length of 53 cm (0.59 s.d.). Her psychomotor development was normal. She spoke first words at 8 month and was able to walk independently at 14 month. She likewise presented with postaxial hexadactyly of the left hand, brachydactyly, a small medial cleft lip, a hypoplastic epiglottis and narrow thorax, in addition we observed a syndactyly VI/VII of the right hand (Supplementary Fig. 1e-g). Her facial feature include a broad and prominent forehead, a depressed nasal bridge, broad and up-slanting nasal tip and low set ears. She also went through respiratory distress syndrome but recovered after 2 month. Beside an episode of pneumonia she showed no signs of increases susceptibility to infections. No further malformation are known.

Patient 3 was the fetus after termination of the pregnancy at 19^th^ week of gestation. During pregnancy polyhydramnion and postaxial hexadacytly was noticed (Supplementary Fig. 1h-j). In addition, the fetus presented with a medial cleft lip and a pronounced narrow thorax.

Radiological investigation of both girls were performed and revealed cone-shaped epiphysis of the phalangeal bones, a metaphyseal dysplasia and a narrow thorax with short ribs (personal communication with Prof. F. Majewksi), so an atypical Jeune syndrome was proposed (Supplementary Fig. 1k,l).

### Whole exome sequencing and candidate screening

Exome sequencing has been demonstrated to be highly successful to identify the underlying genetic cause of autosomal recessive inherited entities^36^,^37^,^38^,^39^,^40^,^41^. After Agilent sure select capturing and exome sequencing of patient 2 we received 156.9 M single end reads. 85.01% of the reads were mapped to the human genome version hg19 and 88.06% were on-target. 81.5% of the captured target was covered at least 5x with an average depth of 73.3-fold. A total of 52,679 variants were called, of which 2,076 were indels and 50,603 were single nucleotide variants. Based on the supposed rare incidence of the phenotype we excluded all frequent variants (above 0.01%), detected in any of the 666 in-house control individuals as well as all annotated variants of dbSNP132, the 1000genomes project (phase1), and the exome variant server (http://evs.gs.washington.edu/EVS). Excluding intergenic, intronic and synonymous variants and proposing an autosomal recessive inheritance led to 58 remaining, variants of unknown significance (VUS) in 4 genes. Based on the mutational effect of these variants, their conservation (PhyloP, GERP++) and prediction program scores of SIFT, PolyPhen2 and MutationTaster, CADD only the two *DYNC2LI1* mutations remained as candidate variants (Supplementary Table 1): A nonsense mutation c.622C > T (p.Arg208Ter; NM_001193464) in exon 8 and the missense mutation c.662C > T (p.Thr221Ile; NM_001193464) in exon 9 of the *DYNC2LI1* gene. Nucleotide numbering uses +1 as the A of the ATG translation initiation codon in the reference sequence, with the initiation codon as codon 1. Both variants could be confirmed by Sanger sequencing (Supplementary Fig. 2) and were not present in 858 control chromosomes of healthy individuals and in the 61,486 exomes of the Exome Aggregation Consortium (Exome Aggregation Consortium (ExAC), Cambridge, MA (URL: http://exac.broadinstitute.org) Nov. 2014). The affected positions are highly conserved on nucleotide and amino acid level. No mutations in any of the known skeletal ciliopathy genes or other potential candidate genes were identified in this individual. The healthy mother, brother and son were carrier for the missense mutation p.Thr221Ile. DNA of the healthy father and the affected patients 2 and 3 was not available. Based on the family history the nonsense mutation is paternally inherited. Therefore, the identified mutations are highly likely to be pathogenic.

### *DYNC2LI1* expression

We were not able to investigate the effect of the mutations on the expression level, as RNA of the patients was not available. We analyzed the expression levels of *DYNC2LI1* in different tissues with adult and fetal complementary DNA (cDNA) panels as well as in osteoblasts and chondrocytes. We examined a ubiquitous expression of *DYNC2LI1* in all examined tissues (Supplementary Fig. 3). However, the overall highest expression levels were measured in chondrocytes, brain and kidney, no differences between fetal and adult tissues were observed except for a higher fetal expression in brain, kidney and lungs. This is in accordance with expression profiles of genes involved in structure and function of the primary cilium. In addition, we observed an expression of all 3 isoforms in the examined tissues with the highest contribution of the 2 longest isoforms.

### Domain structure and structural effects of DYNC2LI1 mutations

**Figure 1:**
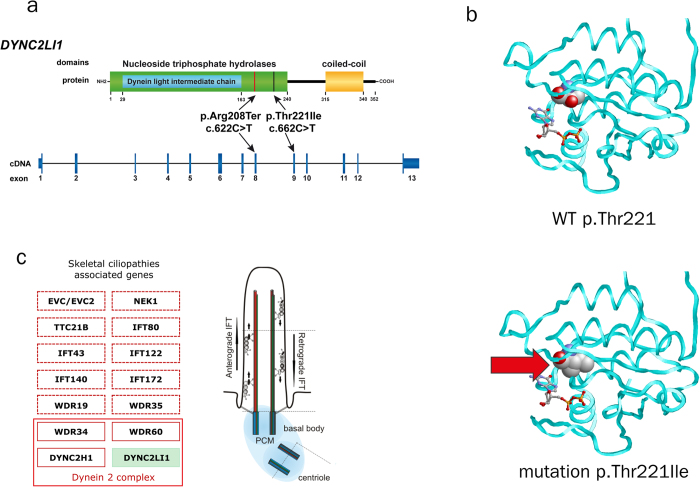
Identified mutations in *DYNC2LI1* and protein model. (**a**) Compound heterozygous missense and nonsense mutation are located in the nucleoside triphosphate hydrolase domain of DYNC2LI1. (**b**) Model of the nucleoside triphosphate hydrolase domain of DYNC2LI1 in the wild-type and p.Thr221Ile mutant. The protein backbone is depicted as cyan tube and residue 221 is shown in space-filled presentation and colored according to the atom types. A bound nucleoside diphosphate (GDP) is shown as sticks. Alternatively, ADP may be bound at this site, which cannot be safely discriminated at the present resolution of the model. (**c**) Scheme of the primary cilium and known mutations in skeletal ciliopathies associated genes (EVC/EVC2 = Ellis-van Crefeld syndrome and WAD; NEK1 = SRTD6; TTC21B = SRTD4; IFT80 = SRTD2; IFT43 = CED3; IFT122 = CED1; IFT140 = SRTD9; IFT172 = SRTD10; WDR19 = SRTD5 and CED4; WDR35 = SRTD7 and CED2; WDR34 = SRTD11; WDR60 = SRTD8; DYNC2H1 = SRTD3; DYNC2LI1 = novel intermediate phenotype of our patients)^25^,^26^,^27^,^28^,^29^,^30^,^34^. Abbreviations: WAD (Weyers acrofacial dysostosis), CED (cranioectodermal dysplasia), SRTD (short-rib thoracic dysplasia).

To date, structural information of DYNC2LI1 remains elusive, but comparison of the protein sequence (UniProt **Q8TCX1-1**) with known motifs predicted a P-loop containing nucleoside triphosphate hydrolases domain (position aa 30 – 240) including a Dynein light intermediate chain domain (position aa 30 – 163; http://www.ebi.ac.uk/interpro) (Fig. 1a). The most common functions of the P-loop containing nucleoside triphosphate hydrolases domains are the hydrolysis of nucleoside triphosphate^42^. Modelling of the nucleoside triphosphate hydrolase domain revealed that Thr221 (Fig. 1a,b) is close to the nucleoside binding site. An exchange from threonine to isoleucine in the mutant causes steric clashes and decreases the site of the nucleoside binding pocket (Fig. 1b). Therefore, the mutation is expected to impair the hydrolase function of DYNC2LI1.

As RNA was not available, we were not able to test whether the nonsense mutation might lead to mRNA decay or a shorter protein. However, a predicted truncated protein will miss the coiled-coil domain (position aa 315 – 340) which might be necessary for interaction with the coiled coil domain of DYNC2H1 of the dynein-2 complex.

### DYNC2LI1 localizes to the primary cilium and DYNC2LI1 defects affect morphology and cilia length

**Figure 2:**
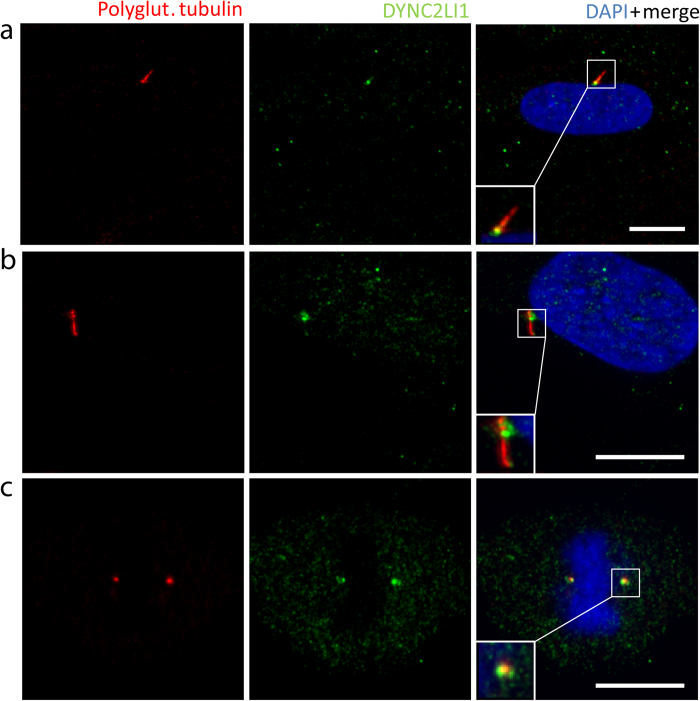
Localization of the DYNC2LI1 protein in control fibroblasts. Immunofluorescence staining of 5 days starved control fibroblasts with antibodies against polyglutamylated tubulin (ciliary axoneme, red), DYNC2LI1 (green) and DAPI (DNA, blue in merge) identified localization of DYNC2LI1 to the basal body region (**a**) and to the transition zone (**b**) of the primary cilium, as well as to the centrosomes (**c**) during mitosis. Magnifications are shown in white boxes, white scale bars 10 μm.

**Figure 3:**
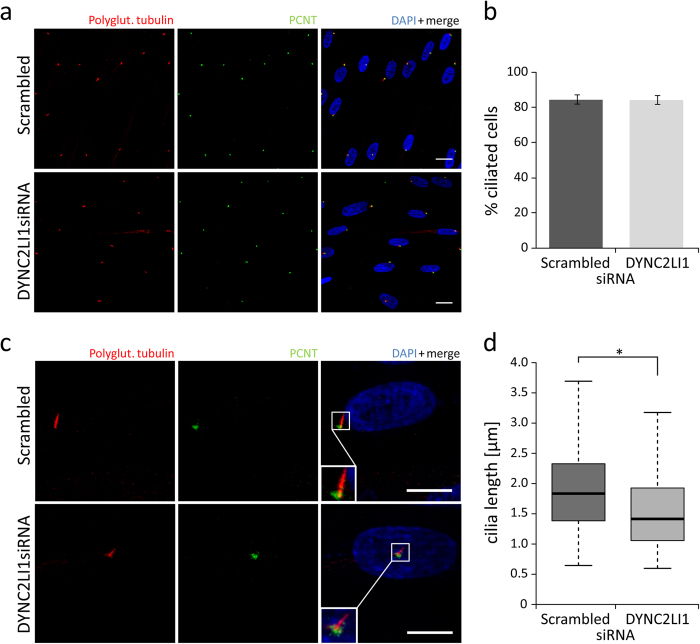
Number of primary cilia and cilia length in *DYNC2LI1* depleted cells compared to scrambled control. Immunofluorescence staining of 5 days starved fibroblasts (scrambled control vs. *DYNC2LI1*siRNA) with antibodies against polyglutamylated tubulin (primary cilium, red), PCNT (green) and DAPI (DNA, blue in merge). (**a,b**) No significant differences in the number of cilium presenting cells were observed, 85% of control cells and 84% of *DYNC2LI1* depleted cells showed primary cilium formation (p = 0.927 [χ2-test], white scale bars 20 μm). (**c,d**) The median cilia length was 1.83 μm in scrambled control cells, whereas cilia in knockdown cells were significantly shorter with median of 1.42 μm (p = 1.205 × 10^−6^ [Mann-Whitney-U test]). Magnifications are shown in white boxes, white scale bars 10 μm).

**Figure 4:**
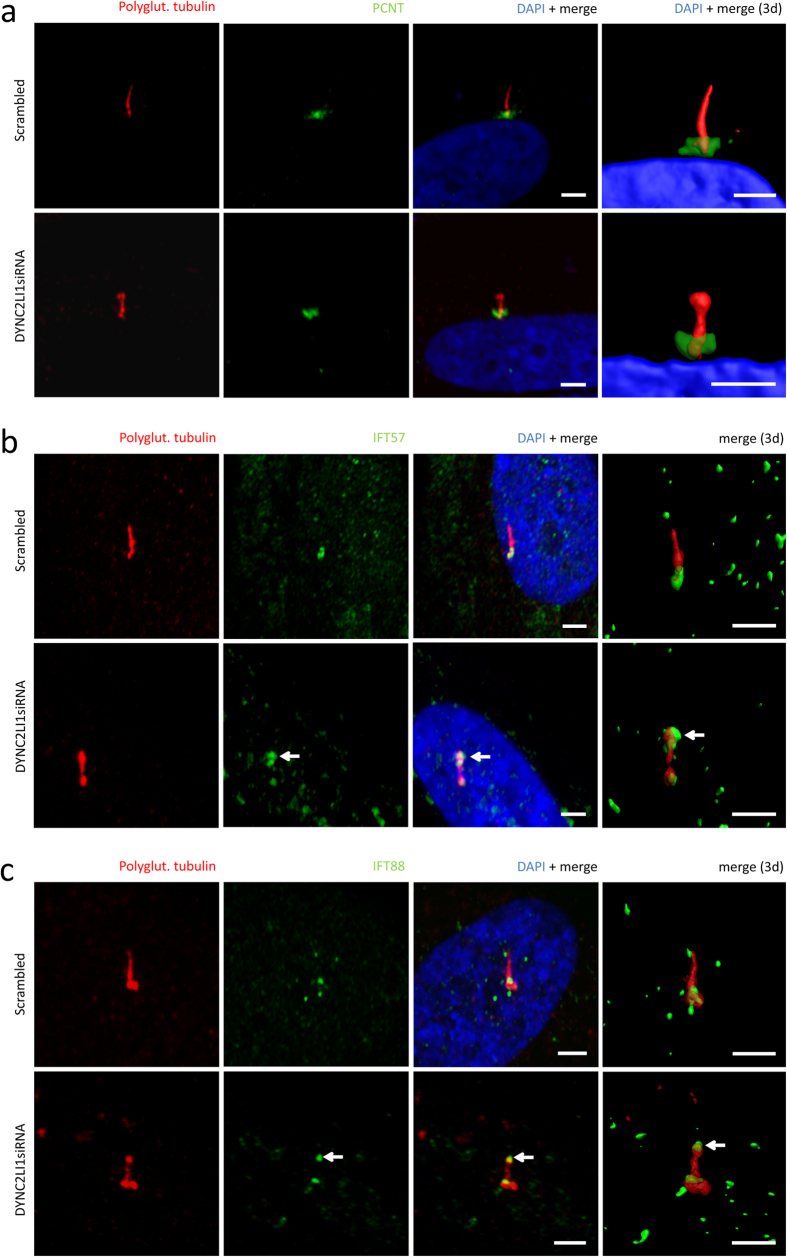
*DYNC2LI1* defect cells present with abnormal cilia morphology confirmed retrograde IFT defects. Immunofluorescence staining of 5 days starved fibroblasts (scrambled control vs. *DYNC2LI1*siRNA) with antibodies against polyglutamylated tubulin (primary cilium, red), PCNT/IFT57/IFT88 (green) and DAPI (DNA, blue in merge). (**a**) *DYNC2LI1* depleted cells showed in addition to the reduced ciliary length further ciliary morphological abnormalities with bulbous tips in 14% of analyzed cilia. Scrambled control cells present normal cilia structure, but we observed as well malformations in 6% of measured control cilia (p = 0.028 [χ2-test]). (**b**) Scrambled controls and knockdown cells showed a localization of IFT57 at the basal body region of the primary cilium. In addition, knockdown of *DYNC2LI1* resulted in an accumulation of IFT57 - component of IFT-B complex - in the bulbous ciliary tip (arrows). (**c**) IFT88 - another component of the IFT-B complex - is localized at the basal body region of the primary cilium in scrambled controls and knockdown cells. IFT88 is present at the ciliary tip in scrambled controls compared to a strong accumulation in the bulbous tip of *DYNC2LI1* depleted cells (arrows). White scale bars 2.5 μm, three-dimensional reconstruction was performed using Imaris software (Bitplane, Zurich).

As the dynein-2 complex is part of the retrograde IFT of the primary cilium we expected the DYNC2LI1 protein to be localized to the primary cilium (Fig. 1c)^35^. Using immunofluorescence analysis in ciliated fibroblast cells we confirmed the localization of the DYNC2LI1 protein to the cytoplasm, centrosomes, as well as around the basal body, and the transition zone of the primary cilium (Fig. 2). To determine the patient’s phenotype on a cellular level we performed immunofluorescence staining on human fibroblasts. As patient-derived fibroblasts were not available, the cellular effects of DYNC2LI1 depletion were examined following siRNA knockdown of *DYNC2LI1* and compared to scrambled siRNA. The remaining expression rate of *DYNC2LI1* was on average 39%. Cilia were examined after siRNA transfection and following 5 days of serum starvation to induce ciliogenesis. While cilia were detected in the majority of the cells, no significant difference in percentage of ciliated cells was detected (Fig. 3 a,b: 85% ciliated cells in scrambled controls vs. 84% in *DYNC2LI1* depleted cells, p = 0.927 χ2-test). As DYNC2LI1 is part of the dynein-2 complex and defects of other components of this complex have been associated with alterations of cilia length and morphology we performed further immunofluorescence analysis of the *DYNC2LI1* depleted cells. Control cilia showed a mean cilia length of median 1.83 μm, in comparison to cilia in *DYNC2LI1* depleted cells, which were only 1.42 μm long (Fig. 3 c,d). Measured differences were highly significant with a p-value of 1.205 × 10^−6^ [Mann-Whitney-U test]. In addition, we observed significantly altered cilia morphology with broadened ciliary tips in 14% of cilia of the *DYNC2LI1* depleted cells, compared to 6% abnormal ciliary structures in scrambled controls [p = 0.028 χ2-test ] (Fig. 4 a). To study the defect in retrograde IFT due to the abnormal ciliary morphology, we stained components of the IFT-B complex IFT57 and IFT88. We observed a localization of these at the basal body region of the primary cilium in scrambled controls and knockdown cells. Morphological abnormal cilia in *DYNC2LI1* depleted cells showed an accumulation of IFT57 and IFT88 in the bulbous ciliary tip confirming a retrograde IFT defect in these cells (Fig. 4 b,c).

## Discussion

The primary cilium has a pivotal role in a variety of complex processes during pre- and postnatal development of almost all vertebrate cells^1^,^14^,^15^. The assembly, maintenance and disassembly of the primary cilium is highly controlled throughout the cell cycle to ensure proper function of the associated pathways^43^. Several disorders have been associated with defects of the primary cilium^15^,^24^,^31^. The observed phenotypes of these ciliopathies represent alteration of the ciliary pathways and can affect nearly every major organ^44^. Thus, these clinical entities have many clinical features in common. This holds true in particular for the group of the skeletal ciliopathies. Here, the most common group constitutes of the short-rib thoracic dysplasias (SRTDs), including the asphyxiating thorax dysplasia / Jeune and Ellis-van Crefeld syndrome^25^,^32^. Jeune syndrome / ATD is distinguished by severely constricted thorax leading to respiratory insufficiency, shortened tubular bones and inconsistent polydactyly^45^,^46^,^47^. Features observed in Elli-van Crefeld syndrome are congenital cardiac defects, dysplastic nails and teeth, and labio-gingival adhesions, as well as skeletal features like polydactyly, short ribs and limbs^48^. These entities show significant clinical overlap and variable phenotypical expression. The affected individuals of the family reported here present with clinical features that include hypoplastic thorax, short stature, polydactyly, hypoplastic nails, and atrioventricular septal defects. In accordance with the taxonomy, our patients have been classified as Jeune-like or EVC.

NGS has been proven to be effective to identify the underlying genetic cause of inherited diseases^36^,^37^,^38^,^39^,^40^,^41^. Concerning the genetic heterogeneity of the skeletal ciliopathies we aimed at the identification of the underlying cause in our family by using whole exome sequencing after SureSelect enrichment. After excluding mutations in the known genes associated with skeletal ciliopathies we focused on rare, coding variants in cilia associated genes^5^,^49^,^50^,^51^,^52^. This identified the two heterozygous mutations c.622C > T (p.Arg208Ter) in exon 8 and c.662C > T (p.Thr221Ile) in exon 9 of the *DYNC2LI1* gene (Fig. 1 a). In agreement with autosomal-recessive inheritance the non-affected family members were heterozygous carriers for the identified mutations. These mutations were confirmed by Sanger sequencing and could be excluded in further control samples. Both mutations were not present in the 61,486 exomes of the Exome Aggregation Consortium and are therefore conserved, rare variants. Mice homozygous for a knockout of the orthologous gene *Dync2li1*^*tm1Aar*^ are not viable and die before embryonic day 11.5^53^. These mice lack monocilia and develop neural tube and cardiac defects, and defects in trunck and tail development. Thus, we considered these mutations as most likely disease causing.

*DYNC2LI1* (*D2LIC*, *LIC3*) consists of 3 isoforms (NM_001193464; NM_016008; NM_015522) with the longest to be composed of 13 exons encoding a 352-amino-acid-long protein. It is ubiquitously expressed with high expression levels in brain, kidney, lung and testes shown in immunoblots of adult mice^54^. In human tissues we confirmed the highest mRNA expression levels in chondrocytes, brain and kidney (Supplementary Fig. 3). Significant difference with higher expression levels in fetal tissues was observed in brain, lung and kidney. The two longest isoforms are predominantly expressed in all examined tissues. As our mutations are located in these 2 longest isoforms only, we considered these to have a pathogenic effect on skeletal development in our patients and therefore to be disease causing.

DYNC2LI1 has been identified as part of the dynein-2 complex involved in the retrograde transport (retrograde IFT)^34^,^35^. Loss of cytoplasmic dynein-2 in *dync2-li1* zebrafish morphants leads to shorter and lower amount of cilia with accumulation of IFT88 near the distal end of the connecting cilium^55^. These morphants have a characteristic phenotype with shortening of the body length, small eyes and kidney cysts in 62% as observed in ciliopathies. Interestingly, defects of other components of the dynein-2 complex, DYNC2H1, WDR34, WDR60 in human have been associated with the clinical phenotype of SRPS type III, Jeune syndrome / asphyxiating thoracic dystrophy (SRTD3/8/11)^34^.

Previous studies localized DYNC2LI1 in mammalian cells at the Golgi apparatus and the centrosomal region^54^,^56^,^57^. In addition, a localization to the axoneme of ciliated epithelia cells was reported. Our immunocytochemistry using an antibody against DYNC2LI1 supported the localization to the centrosome (Fig. 2c). Moreover, we refined the reported localization in the axoneme to be concentrated at its base and at the region of the transition zone (Fig. 2a,b). Even though Madhivanan *et al*.^58^ speculated that defects of proteins involved in the control of vesicle trafficking to the cilium leads to a more severe phenotype than defects of the IFT only, the pronounced clinical phenotypes of patients with dynein-2 complex defects, Jeune and our patient, do not support this observation.

On the basis of the observed ciliary defects caused by dysfunction of IFT and basal body proteins, we expected loss of function of DYNC2LI1 to cause morphological abnormal and missing cilia in humans. As we were not able to obtain patient cells, we used immunofluorescence in *DYNC2LI1* depleted human fibroblasts. We did not observe a significant difference in the percentage of ciliated cells (Fig. 3a,b), a significant average size reduction could be detected (Fig. 3c,d). The observation of shorter cilia confirmed the results in zebrafish morphants and *Dync2li1*^*tm1Aar*^ knockout mice^53^,^55^. Reduced cilia length with bulbous tip was although reported for defects of DYNC2H1 and WDR34, other components of the dynein-2 complex^28^,^59^. However, one report showed no significant difference in proportion of ciliated cells and cilia length in *Wdr34*-knockdown ATDC5 lines^29^. In a further component of the dynein-2 complex, WDR60, a reduced amount of ciliated cells but normal cilia length was observed in cell lines of affected patients^30^. Together, these data indicate that the reduction of ciliated cells as well as of cilia length is a common hallmark of dynein-2 defects. In addition, we showed a higher incidence of cilia with a bulbous tip suggesting a defect in microtubule architecture (Fig. 4 a). This was also observed in *Dync2li1*^*tm1Aar*^ knockout mice where stumpy nodal cilia contain disorganized microtubules, IFT proteins and cellular debris^53^,^60^. IFT-B proteins to be accumulated in cilia with retrograde IFT transport defects include IFT57 and IFT88^30^,^61^. Morphological abnormal cilia in our knockdown cells showed a significant accumulation at the ciliary tip confirming the IFT transport defect in *DYNC2LI1* deficient cells (Fig. 4 b,c).

As primary cilia act as sensors for different pathways, the defect of the dynein-2 complex leading to retrograde IFT impairment is consistent with impairment of these signaling cascades. Among others, defects of the Sonic Hedgehog pathway have been associated with limb-development defects confirming the skeletal phenotype in our patients^24^,^62^,^63^.

In conclusion, mutations in members of the dynein-2 complex result in Jeune syndrome / ATD and other forms of the SRTD spectrum. Different defects of this complex suggest a correlation between the amount of ciliated cells, the resulting cilia length and the functional IFT components with the phenotypically overlapping, but variable clinical features. Thus, the identification of mutations in *DYNC2LI1* in our patients expands the phenotypic spectrum of skeletal ciliopathies.

## Methods

### Subjects and clinical data

Informed consent was obtained from the patients for experimental protocols and displaying participants’ facial appearances in publications. Peripheral blood samples were obtained from the patients as well as control individuals. Genomic DNA from all collected samples was extracted according to standard procedures. This study was approved by the Ethical Review Board of the Friedrich-Alexander-Universität Erlangen-Nürnberg.

### Whole Exome Sequencing

DNA of patient 2 was enriched using the Agilent’s SureSelect Human All Exon Kit V3 (Agilent technologies, Santa Clara, CA) and sequenced in single-end reads on a SOLiD system (Life Sciences, Santa Clara, CA). Sequenced reads were mapped with LifeScope (Life Technologies, Carlsbad, CA) to the reference human genome assembly hg19 (GRCh37). Genotypes were additionally called with GATK 2 and SAMtools^64^,^65^,^66^. For variant annotation ANNOVAR^67^ (http://www.openbioinformatics.org/annovar) was used to check on gene information, substitutions of amino acids, predictions of SIFT v. 1.03 (http://sift.jcvi.org/)^68^, PolyPhen2 (http://genetics.bwh.harvard.edu/pph2/)^69^ and MutationTaster (http://www.mutationtaster.org/)^70^, entries of dbSNP132, as well as allele frequencies of the 1000 Genomes Project (http://www.1000genomes.org)^71^ and NHLBI-ESP 5400. An in-house database consisting of 666 sequenced exomes was also used to exclude sequence errors and frequent variants. Graphical presentation of the mapped sequences was viewed with the Integrative Genomics Viewer (IGV)^72^,^73^. Variants were selected based on autosomal recessive inheritance, their population frequency, the location within the coding sequence (exon, splice site) and the functional effect. The resulting variants were Sanger sequenced and the segregation in the family tested

### Protein modelling

The domain architecture of DYNC2LI1 was investigated using the InterPro^74^ and Genesilico^75^ Meta prediction servers. Molecular modelling of residues 28-241 of DYNC2LI1 was performed with Modeller 9.9^76^ using the crystal structure of human RAB5B in complex with GDP (PDB code: 2HEI) as a template. The mutation was modeled using SwissModel^77^ and RasMol was used for structure analysis and visualization^78^.

### *DYNC2LI1* expression analysis in human tissues

Relative *DYNC2LI1* expression was measured by quantitative real-time PCR using predesigned TaqMan Gene Expression Assays with the TaqMan Gene Expression Mastermix (Life Technologies) specific for all 3 isoforms (Hs00602913_m1, spanning exon 5-6), isoform 4 (Hs01005273_m1, spanning exon 6-7 of NM_001193464) and isoform 1 (Hs01011588_m1, spanning exon of 6-7 NM_016008). An isoform 2 (NM_015522) specific probe was designed using the Custom TaqMan Assay Design Tool (Life Technologies) for the target region chr2:44021785-44022314 (3’ UTR).

Expression levels were quantified in quadruplicates for each gene using the ΔΔCt method with four endogenous control genes (Beta-Actin [huACTB], Beta-2-Microglobulin [huB2M], acidic ribosomal protein [huPO], phosphoglycerate kinase 1 [huPGK1]).

We used commercially available cDNA panels (Human Fetal MTC^TM^ Panel”, “Human MTC^TM^ Panel I” [Clonetech, Mountain View, USA]) covering fetal (whole brain, heart, liver, lung, kidney) and adult (whole brain, heart, liver, lung, kidney) tissues to determine the expression pattern of *DYNC2LI1* in different human tissues. RNA of osteoblasts and chondrocytes were obtained from 2 different (osteoblasts) and 8 different (chondrocytes) adult individuals.

### Cell culture

Primary fibroblasts of a healthy control were cultured using Dulbecco’s modified Eagle medium (DMEM, Life Technologies), patients fibroblasts were not available. Supplements were 0–10% fetal calf serum (FCS, Gibco) depending on the experiment and 1% antibiotic mixture (Penicillin-Streptomycin-Glutamine; Life Technologies). Transfection of *DYNC2LI1* siRNA (HSS147516; Life Technologies) was carried out in medium without antibiotics using Lipofectamin 2000 (Life Technologies) according to the manufacturer’s instructions. Ciliogenesis was induced by incubating cells in serum-free medium (0% FCS) for a period of 5 days.

### *DYNC2LI1* knockdown

Stealth RNAi™ siRNA Negative Control and 3 *DYNC2LI1* Stealth RNAi™ siRNAs (HSS182153, HSS147514, HSS147516) were purchased from Life Technologies. The *DYNC2LI1* siRNA HSS147516 with the lowest level of residual activity in control experiments and the corresponding Stealth RNAi™ siRNA Negative Control Lo GC (scrambled control 12935-200) were used.

### Immunofluorescence

For immunofluorescence analyses cells were fixed with an EGTA-saturated ice-cold 100% methanol solution. Cells were treated with 0.01% Tween for 10 min followed by blocking of non-specific protein interactions for 60 min with blocking solution (0.5% cold-water fish gelatin and 0.1% BSA in PBS). Incubation with primary antibodies (diluted in blocking solution) was performed overnight at 4° C. Centrosomes or the basal body of the primary cilium were stained with MmPeriC1 (rabbit, anti-Pcnt pAb, 1:500,^79^) and the primary cilium was stained with GT335 (mouse, anti-Polyglutamylation Modification mAb, 1:1000, AdipoGen, San Diego CA, USA). For DYNC2LI1 localization studies we used anti-DYNC2LI1 (rabbit, 15949-1-AP, 1:100, Acris, Herford, Germany). IFT57 (rabbit, 11083-1-AP, 1:100, Proteintech, Manchester, United Kingdom) and IFT88 (rabbit, 13967-1-AP, 1:200, Proteintech, Manchester, United Kingdom) antibodies were used for accumulations studies of IFT-B proteins in ciliary tips. Alexa Fluor® 488 goat anti- rabbit IgG (green, 1:500) and Alexa Fluor® 594 goat anti-mouse IgG (red, 1:500) (Life Technologies) were used as secondary antibodies and cell nuclei were stained with DAPI (4′,6-Diamidin-2-phenylindol, 1:50000, Serva, Heidelberg, Germany). Samples were mounted in Aqua Poly Mount (Polysciences, Eppelheim, Germany) and analyzed with a Zeiss Axio Imager Z2 fluorescence microscope equipped with an Apotome. Projections of z-stacks were calculated with AxioVision 4.8 software (Zeiss, Oberkochen, Germany) and the images were adjusted for brightness and contrast using Adobe Photoshop CS (Adobe, San Jose, CA). The length of primary cilia was measured with the FIJI program^80^.

We performed three-dimensional reconstruction of cilia using Imaris software 7.7 (Bitplane, Zurich, Switzerland) with picture stacks from a Laser Scanning Microscope 710 (Carl Zeiss) and ZEN 2010 software with corresponding imaging modules.

## Additional Information

**How to cite this article**: Kessler, K. *et al*. *DYNC2LI1* mutations broaden the clinical spectrum of dynein-2 defects. *Sci. Rep*. **5**, 11649; doi: 10.1038/srep11649 (2015).

## Supplementary Material

Supplementary Information
