# Supplementary material for: DYNC2LI1 mutations broaden the clinical spectrum of dynein-2 defects
Source: Sci Rep. 2015 Jul 1;5:11649. doi: 10.1038/srep11649 (PMC4486972; doi:10.1038/srep11649)
Supplement: Supplementary Information [file srep11649-s1.doc]

**Supplementary Information**

***DYNC2LI1* mutations broaden the clinical spectrum of dynein-2 defects**

Kristin Kessler^1^, Ina Wunderlich^1^, Steffen Uebe^1^, Nathalie S Falk^2^, Andreas Gießl^2^, Johann Helmut Brandstätter^2^, Bernt Popp^1^, Patricia Klinger^3^, Arif B Ekici^1^, Heinrich Sticht^4^, Helmuth-Günther Dörr^5^, André Reis^1^, Ronald Roepman^6^, Eva Seemanová^7^, Christian T Thiel^1*^

^1^ Institute of Human Genetics, Friedrich-Alexander-Universität Erlangen-Nürnberg, Erlangen, Germany

^2^ Animal Physiology, Friedrich-Alexander-Universität Erlangen-Nürnberg, Erlangen, Germany

^3^ Department of Orthopaedic Rheumatology, Friedrich-Alexander-Universität Erlangen-Nürnberg, Erlangen, Germany

^4^ Institute of Biochemistry, Friedrich-Alexander-Universität Erlangen-Nürnberg, Erlangen, Germany

^5^ Department of Pediatrics and Adolescent Medicine, Friedrich-Alexander-Universität Erlangen-Nürnberg, Erlangen, Germany

^6^ Department of Human Genetics, Radboud University Medical Center, Nijmegen, Netherlands

^7^ Department of Clinical Genetics, Institute of Biology and Medical Genetics, 2nd Medical School, Charles University, Prague, Czech Republic

**Table of contents**

**Supplementary Figures**

Supplementary Figure 1. Morphological and radiographic features of patients with *DYNC2LI1* mutations.

Supplementary Figure 2. Visualization of the *DYNC2LI1* mutations.

Supplementary Figure 3. *DYNC2LI1* expression pattern in different adult and fetal tissues.

**Supplementary Tables**

Supplementary Table 1. Exome candidate variants


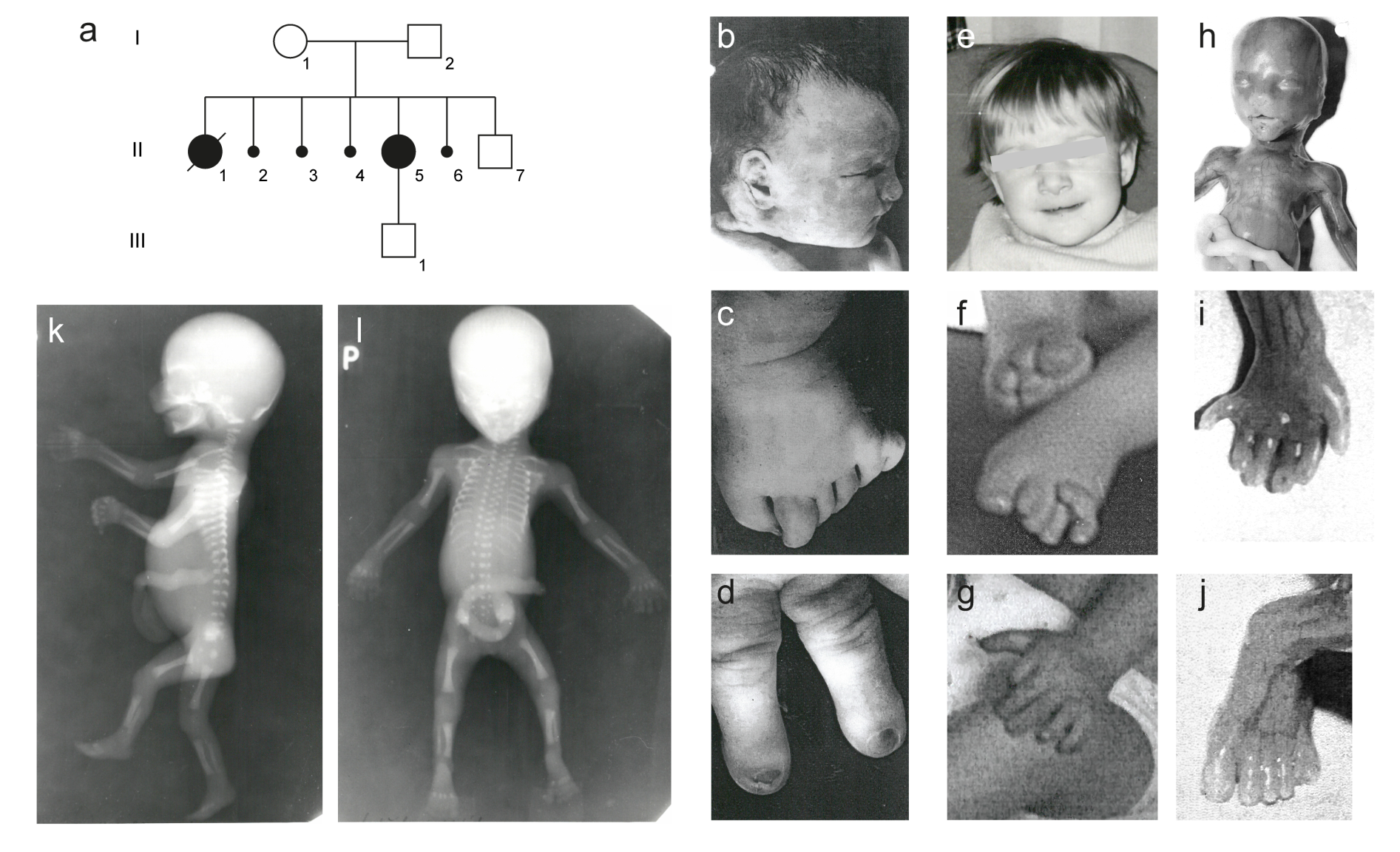


**Supplementary Figure 1.** **Morphological and radiographic features of patients with *DYNC2LI1* mutations.** (**a**) Pedigree of the family. (**b-d**) Facial gestalt of patient 1 (II.1), postaxial hexadactyly and nail dystrophy. (**e**) Facial gestalt of patient 2 (II.5) with broad and prominent forehead, a depressed nasal bridge, broad and up-slanting nasal tip and low set ears. (**f-g**) Patient 2 presenting postaxial hexadactyly. (**h-j**) Patient 3 (II.6) 19^th^ week of gestation showed medial cleft lip, pointed chin, narrow thorax and postaxial hexadactyly. (**k,l**) Radiographic features of patient 3 include long tibia, metaphyseal dysplasia and short ribs.


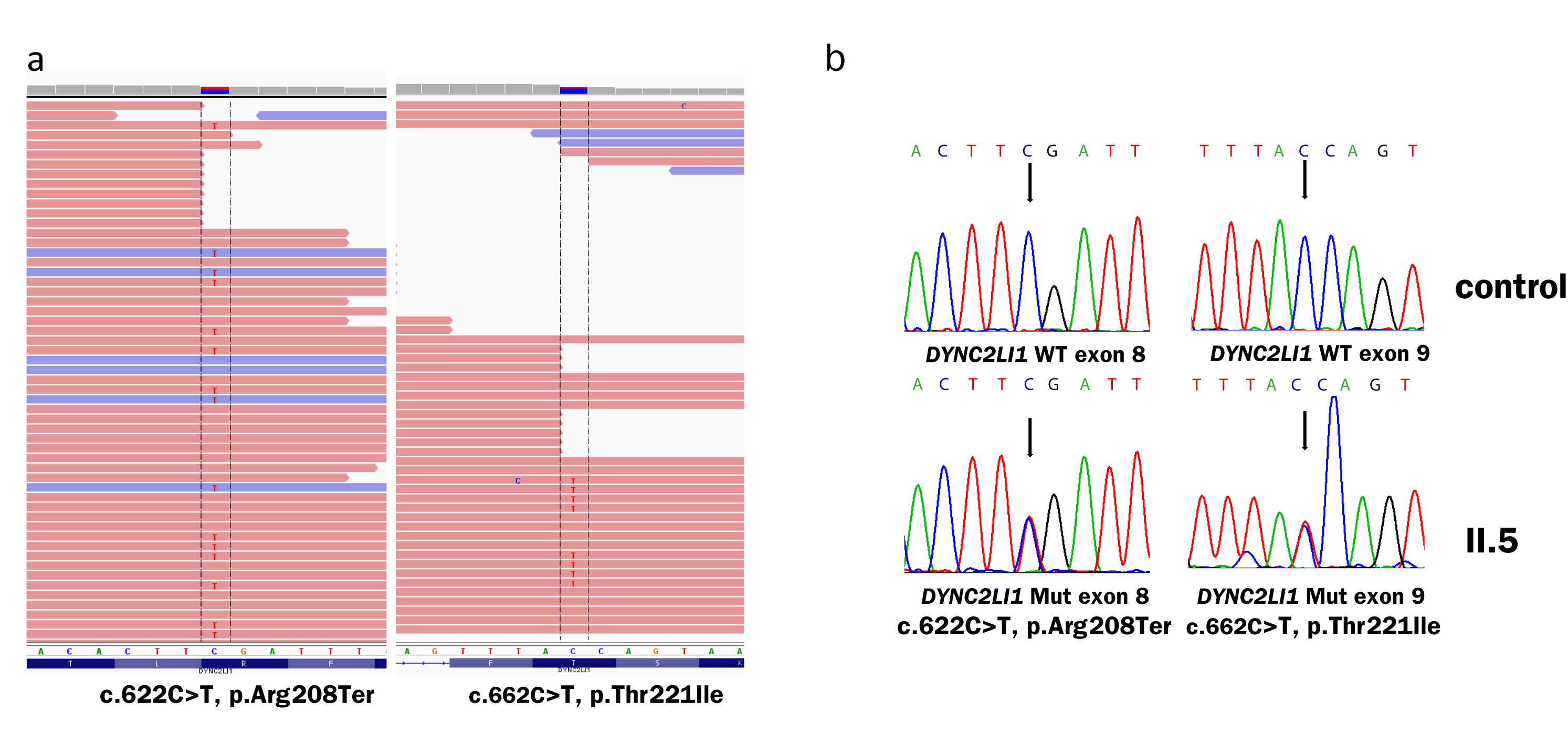


**Supplementary Figure 2. Visualization of the *DYNC2LI1* mutations.** (**a**) IGV browser presentation of both mutations in the *DYNC2LI1* gene detected by Next Generation Sequencing. (**b**) Sanger confirmation in the affected patient II.5.


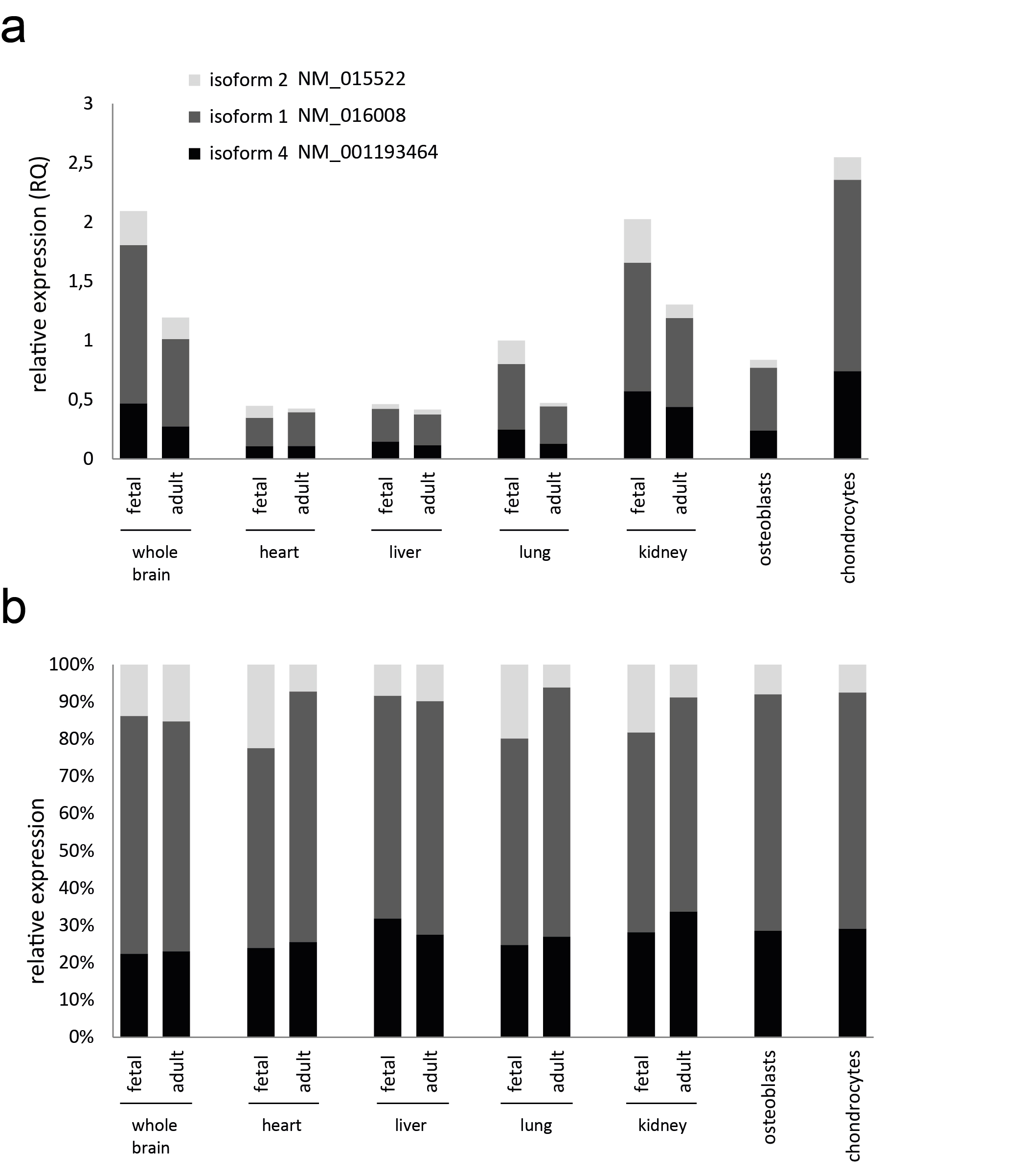


**Supplementary Figure 3. *DYNC2LI1* expression pattern in different adult and fetal tissues.** (**a**) Relative expression levels of DYNC2LI1 of all three isoforms (isoform 1 and 4 consists of 13 exons, isoform 2 of the first 6 exons with an alternative 3’UTR). Note the highest expression levels in chondrocytes. (**b**) The percentage of relative isoform expression is shown in relation to overall expression.

**Supplementary Tables**

| **Supplementary Table 1. Exome candidate variants** | | | | | | | | | | | |
| --- | --- | --- | --- | --- | --- | --- | --- | --- | --- | --- | --- |
| Gene | Exon | Position | RefSeq | cDNA | Protein | PhyloP | GERP++ | SIFT score | PolyPhen2 score | MutationTaster prediction | CADD |
| *ADAM7* | 3 | chr8 :24304721 | NM_003817 | c.179T>C | p.Leu60Ser | 0.974 | 3.15 | 0.121 | 0.653 | polymorphism | 9.923 |
| *ADAM7* | 19 | chr8 :24358382 | NM_003817 | c.2082A>C | p.Lys694Asn | -1.322 | -6.53 | 0.218 | 0.004 | polymorphism | 4.688 |
| ***DYNC2LI1*** | **8** | **chr2 :44023899** | **NM_001193464** | **c.622C>T** | **p.Arg208Ter** | **2.662** | **5.13** | **na** | **na** | **disease causing** | **39** |
| ***DYNC2LI1*** | **9** | **chr2 :44027984** | **NM_001193464** | **c.662C>T** | **p.Thr221Ile** | **2.639** | **5.09** | **0.079** | **0.068** | **disease causing** | **15.06** |
| *MUC17* | 3 | chr7 :100680255 | NM_001040105 | c.5558A>C | p.Glu1853Ala | 0.655 | 0.824 | 0.047 | 0.168 | polymorphism | 3.13 |
| *MUC17* | 3 | chr7 :100682379 | NM_001040105 | c.7682G>T | p.Gly2561Val | -0.565 | -1.91 | 0.285 | 0 | polymorphism | 0.024 |
| *MUC4* | 2 | chr3 :195515221 | NM_018406 | c.3230G>A | p.Ser1077Asn | -1.019 | -1.66 | 0.306 | 0.003 | polymorphism | 5.93 |
| *MUC4* | 2 | chr3 :195515231 | NM_018406 | c.3220C>A | p.Pro1074Thr | -1.347 | -2.01 | 0.068 | 0.307 | polymorphism | 4.265 |
